# REMOTE, a Wireless Sensor Network Based System to Monitor Rowing Performance

**DOI:** 10.3390/s90907069

**Published:** 2009-09-04

**Authors:** Jordi Llosa, Ignasi Vilajosana, Xavier Vilajosana, Nacho Navarro, Emma Suriñach, Joan Manuel Marquès

**Affiliations:** 1 Universitat Oberta de Catalunya, Rambla Poblenou 156 08018 Barcelona, Spain; E-Mails: xvilajosana@uoc.edu (X.V.); jmarquesp@uoc.edu (J.M.M.); 2 WorldSensing S.L.N.E, Adolf Florensa 8, 08028 Barcelona, Spain; E-Mail: ivilajosana@worldsensing.com; 3 Universitat Politécnica de Catalunya, Jordi Girona 1, 08034 Barcelona, Spain; E-Mail: nacho@ac.upc.edu; 4 Universitat de Barcelona, Martí i Franquès, s/n, 08028 Barcelona, Spain; E-Mail: emma.surinach@ub.edu

**Keywords:** motion sensor, 6 degree motion, accelerometer, wireless sensor network, sports performance analysis

## Abstract

In this paper, we take a hard look at the performance of REMOTE, a sensor network based application that provides a detailed picture of a boat movement, individual rower performance, or his/her performance compared with other crew members. The application analyzes data gathered with a WSN strategically deployed over a boat to obtain information on the boat and oar movements. Functionalities of REMOTE are compared to those of RowX [1] outdoor instrument, a commercial wired sensor instrument designed for similar purposes. This study demonstrates that with smart geometrical configuration of the sensors, rotation and translation of the oars and boat can be obtained. Three different tests are performed: laboratory calibration allows us to become familiar with the accelerometer readings and validate the theory, ergometer tests which help us to set the acquisition parameters, and on boat tests shows the application potential of this technologies in sports.

## 1. Introduction

Wireless Sensor Networks (WSN) have been applied to gather data from a large number of scenarios with various purposes such as environmental monitoring [2], structural monitoring [3] or movement tracking [4]. Most of the efforts concentrated on the field of data acquisition and subsequent data analysis for scientific studies. WSN provide the potential to collect data at spatial and temporal scales that are often times not feasible with existing instrumentation. Indeed, deployment of low cost WSN have been proven to be adequate mechanisms for on the field data acquisition, but little has been done using WSN as infrastructure for sports oriented commercial applications.

With the rapid advances in sports technologies, athletes and sports coaches are constantly searching for improved performance assessment methods. Whilst athletic performances continue to improve, accurate training prescription and feedback is important to the consistency of the training outcome and maintaining the performance margin. WSN turn out to be miniaturized wireless sensors that provide real-time feedback and in situ analysis of the biomechanics indices of the athletes during training.

In this paper, we take a hard look at the performance of REMOTE, a sensor network based application that provides a detailed picture of a boat movement, individual rower performance, or his/her performance compared to other crew members. The application analyses data gathered with a WSN strategically deployed over a boat to obtain information on the boat and oar movements. The functionalities of REMOTE are compared to those of RowX [1] outdoor instrument, a commercial wired sensor instrument designed for similar purposes.

## 2. Background and Motivation

The objective of athletes is to improve their performance to achieve their objectives. Athletes improvement depends on multiple metrics that coaches try to study, correlate and understand. Normally, most of the metrics are studied through simple observation or athletes sensations. These information, even extremely useful, is not accurate or completely objective. One of the main problems is to obtain objective diary information of the capacities and abilities of the athletes, without interfering with them.

In this paper we focus on rowing, as an example of sport where rowing technique plays a crucial role to improve rowers performance. In this work, we show how information related to the rowing technique can be gathered in an unprecedented way and used to track oars and boat motion, monitor the balance of power transmitted by both arms, or monitor the equilibrium and balancing of the athlete during the effort, and objectively determine the endurance of the athlete. Moreover, the obtained information could be used to follow rower evolution during a season, or to compare its performance to ideal cases or other rowers using the same system.

Nowadays, RowX [1] is the most advanced commercially available device for daily use that has been specially designed to gather information from rowers. RowX, is a complete system which allows to measure, force exerted on each oar, rotation angle of each oar, boat speed, boat acceleration in X and vertical (Z) axis.

However, the wired infrastructure make it bulky and difficult to install and uninstall, which limits its frequency of use to sporadic sessions of training or tests. It is clear that frequent measurements are essential to track athletes evolution but the system may not interfere with the athlete. The later is one of the drawbacks of the RowX system: due to its visibility, athletes know that they are being measured and consequently don’t behave as if they were not measured, introducing some distortion in the results. Moreover, it is essential to keep real time monitoring of the athlete performance which is not achieved with RowX system, where the data has to be downloaded after the test.

In this work we try to achieve similar results as RowX with a new approach based on wireless sensor networks (WSN). The REMOTE system is based on miniaturized motion sensors (accelerometers) with the ability to communicate wirelessly. The system covers the requirements of monitoring athletes performance in real time with three main advantages respect RowX: (a) freedom of motion due to its wireless characteristics, (b) easy installation and non invasive measurements due to its reduced size, and (c) immediate feedback.

## 3. System Architecture

### 3.1. Network Hardware

**Figure 1.**
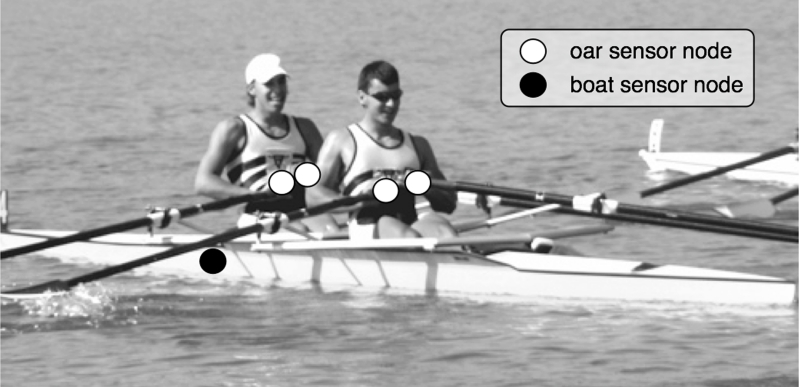
Position of the sensor nodes on the boat.

A minimum REMOTE system is constituted by 3 different elements (Figure 1): Oar sensor nodes that allows local monitoring of the strokes, boat sensor nodes responsible of monitoring boat motion, and the base station that collects the sensed data.

The core of each sensor node is based on telosb platform [5]. The telosb is used as a node on the wireless sensor network. It features a Texas Instruments MSP430 microcontroller, 48 Kbytes of program memory, 10 Kbytes of static RAM, 1Mbyte of external flash memory, and a 2.4 GHz Chipcon CC2420 IEEE 802.15.4 radio. The telosb was designed to run TinyOS [6]. We chose telosb because MSP430 microprocessor provides several configurable ports that easily support external devices. The large amount of flash memory is useful for buffering collected data, as described below.

In order to monitor oar motion we examined the feasibility of designing an accelerometer-based sensor that uses only accelerometers to compute the linear and angular motions of a rigid body. The main motivation for developing a gyroscope-free system was that low-cost gyroscopes lack the accuracy needed for precise oars monitoring [7]. Moreover, low cost gyroscopes (automotive applications) cost about an order of magnitude more with respect of 3 axis accelerometers. In addition, it was reported in [8, 9] that due to challenges associated with gyroscopes hardware integration, the levels of precision required for our problem cannot be achieved in the near future with inexpensive gyroscopes.

**Figure 2.**
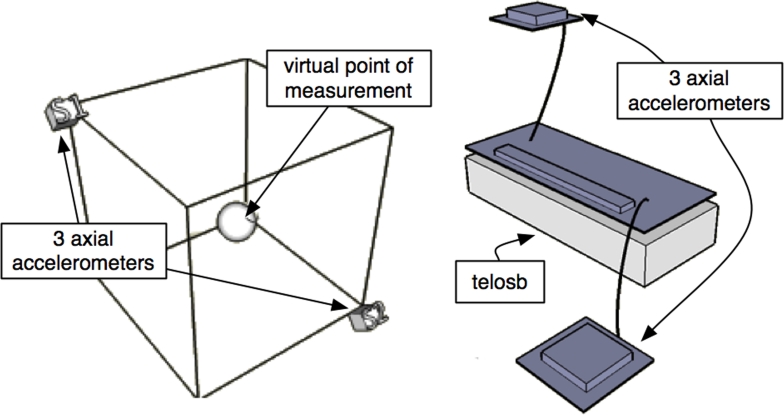
Diagram of the accelerometers position.

For the later reasons, we were limited to use accelerometers by our prime design constrain which was minimize sensors cost. In theory, a minimum of six accelerometers is required for a complete description of a 3D rigid body motion [10, 11]. The key to a solution of the feasibility problem is the choice of location and orientation of the accelerometers. In our case a feasible cube configuration of six accelerometers is then considered (Figure 2), and based on this configuration, a basic algorithm to subtract rotation and translation was developed.

**Figure 3.**
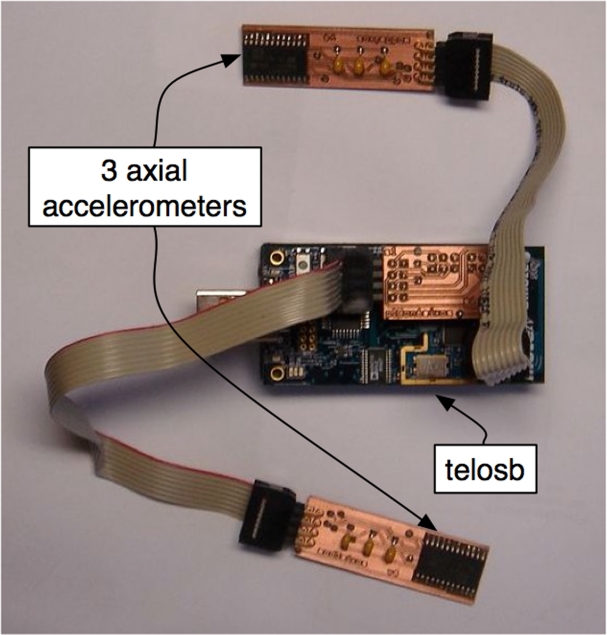
Developed PCB plugged to telosb.

To adapt two MEMS (Micro Electro Mechanical System) accelerometers to the required geometry without jeopardizing system flexibility, two sensor boards for each sensor node, as presented in Figure 3, were developed. Each board was equipped with a LIS3L02AS4 [12] MEMS low-power three axes linear accelerometer.

The LIS3L02AS4 has a user selectable full scale range of *±*2*g*, *±*6*g* and it is capable of measuring accelerations over a bandwidth of 1.5KHz for all axes. It features a signal conditioning, a 1 pole low pass filter at 100 Hz, and temperature compensation. It has an analog output which is sampled using the microcontrollers embedded 12 bit AD converter. The power consumption of the LIS3L02AS4 is less than 0.85 mW [12].

**Figure 4.**
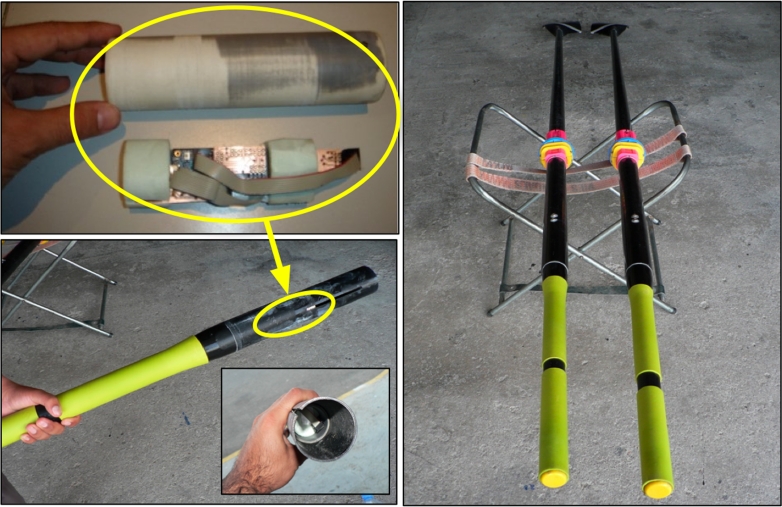
Mounting the mote inside the oar.

A single sensor node consisted of the sensor board, the interface board and battery holder with two AA batteries, all of which were housed inside a cylindrical PVC tube weatherproof and watertight, as presented in Figure 4, which was adapted to the inside oar end.

The boat sensor node was also equipped with a two single three axis accelerometer to track vertical and horizontal motion of the boat. The difference among oar and boat sensors was the packaging. In that case, a waterproof case to keep the sensor dry from water splashing replaced the cylindrical packaging used for the oar sensors. As a base station we used a telosb mote connected to a laptop which could be used in the same boat or in the accompanying boat.

### 3.2. Software of the Nodes

REMOTE system was based on telosb platform [5]. All the software was developed using tinyOS 2.x [6] operating system. TinyOS is a wireless sensor networks specific operating system which follows an event-driven programming model. The programming language is nesC, which is an extension of C with additional concept of components and bidirectional interfaces. The components allow programmers to encapsulate the behavior of the different parts of the program independently. The communication between the components is achieved by the use of bidirectional interfaces. This approach eases collaboration between different programmers, ensures cleaner code, and eases reutilization.

For oars and boat nodes, a tinyOS based program was developed. As specified by tinyOS programming model, a component for each tasks is defined. Components are connected to achieve the expected behavior of the system. A *message manager* component manages the main function of the system. Specifically, the *message manager* component is responsible of the communication between the motes and the base station. All other components are connected to it. The *message manager* decodes received messages and triggers the correct action. The main functions of this component are turning on/off data sampling, setting sampling rate, sending sampled data, and sending information messages to the base station. A *data collector* component collects the data from the accelerometer driver. This driver is a component capable of acquiring the data from the six accelerometers using the ADC component built in tinyOS. Another important aspect is the time synchronization; in order to compare the data obtained from different nodes of the network a global basetime is required. Therefore, a component that implements the FTSP time synchronization protocol [13] was used. This component permits the timestamping of the data. This allows the correlation of data between nodes. In addition, during the collection of data, in order to save data timestamps and accelerometer readings, a buffer is used. This buffer is stored into an on-board flash memory.

The base station program acts as a gateway between the wireless sensor network and the on board laptop. On the computer side, a program coordinates the system: system start/stop, and data collection (oars and boat nodes). When the base station receives the order to retrieve the data, it simply sends a message to each mote, and the message manager bulks the data wirelessly to the laptop. The collected data is stored in a comma-separated format for subsequent analysis.

## 4. Data Analysis and Results

One of the goals of this paper is to validate the utility of WSN for improving elite rowing performance. Nowadays this goal is achieved by subjective observations of the trainer, who corrects the movement of the rowers from a neighboring boat. However this observations are subjective and many times imprecise. More sophisticated techniques in this field include bulky wired systems, they are usually very expensive, heavy, power hungry and difficult to install.

### 4.1. Remote Analysis

Prior to the installation of the system to a boat, a test phase was carried out in which we characterized sensors output. The analog outputs from the used accelerometers are called ratiometric, meaning that they output a voltage proportional to the acceleration seen. For example, when using a 12-bit ADC, where the output values range between 0 and 4096, a 0g reading should correspond to a sampled value of 2048, exactly in the middle of the range. However, in reality, this is not the case, as process variation and other factors making the 0 g position to be different for all sensors. Also, the amount of voltage change for a given change in sensed acceleration is not constant for all sensors. In order to fully characterize our sensors, we performed a 0 calibration to each sensor following a method which involved rotating the accelerometer 180 degrees about the axis perpendicular to the desired axis of calibration and taking the highest and lowest readings the 0 g acceleration (*B̂*) and the sensitivity (*Â*) were determined for each axis of each sensor using Equation 1:

(1) $$\hat{A}=\frac{{\hat{a}}_{\max}-{\hat{a}}_{\min}}{2};\hat{B}=\frac{{\hat{a}}_{\max}+{\hat{a}}_{\min}}{2}$$

Moreover, accelerometer readings measure the force, per unit mass, acting on the body along a specific sensing direction. This force vector is also known as the specific force *F̂*. The vector sum of this force and the gravity *ĝ*, per unit mass is the inertial acceleration of the body. That is:

(2) $$\hat{a}=\hat{F}+\hat{g}$$

In order to subtract the specific force, *F̂* from the acceleration readings we took advantage of the geometrical configuration of the sensors. For each axis we could subtract gravity effects considering that:

(3) $${\hat{a}}_{s1}=\hat{F}+\hat{g}\cdot {\cos\theta }_{1};{\hat{a}}_{s2}=\hat{F}+\hat{g}\cdot {\cos\theta }_{2}$$

**Figure 5.**
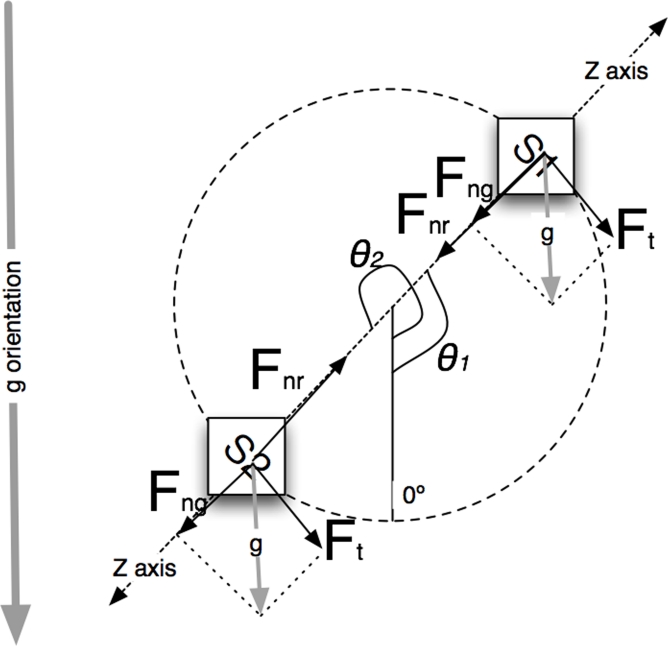
Diagram of forces.

Where *â_si_* is the accelerometer reading, *F̂* is the desired specific acceleration, *ĝ* corresponds to the gravity component and *θ_i_* is the inclination angle respect the vertical (Figure 5) and that the two sensors S1 and S2 present complimentary orientations:

(4) $${\cos\theta }_{1}=-\cos\left(\theta_{2}\right)$$

Combining Equations 3 and 4 We obtain for the specific acceleration:

(5) $$\hat{F}=\frac{{\hat{a}}_{s1}+{\hat{a}}_{s2}}{2}$$

**Figure 6.**
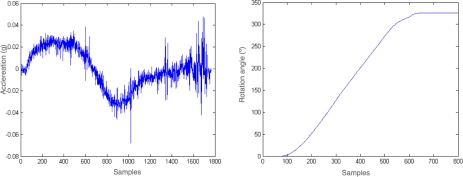
Extraction of the rotation of a rotated object.

To proof the validity of this equation, we tested the performance of the system to obtain pure rotations. Specifically, the accelerometers were installed in a cylindrical tube which was rotated 360 degrees. In Figure 6 the accelerometers readings and the extracted rotation angle for this experiment are presented (Equation 6).

(6) $$\theta -\theta_{0}=\int \sqrt{\frac{\hat{F}}{\hat{R}}}\mathrm{dt}$$

**Figure 7.**
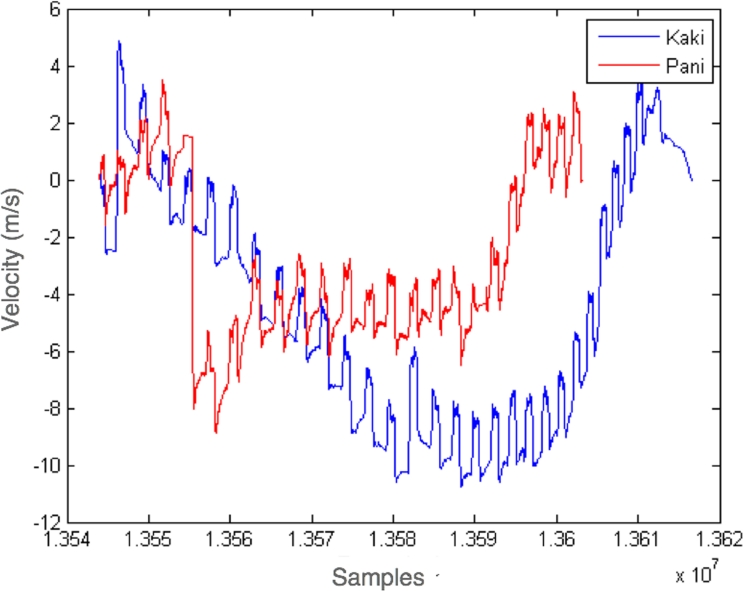
Velocity evolution of two sportsmen at ergometer

To verify the performance of the entire system, two additional experiments were carried out. In the first approach, for simplicity, we monitored two athletes in a ergometer workout, the later helped to produce the first “rowing-related” sets of results. Specifically, we were interested in monitoring the forces exerted by the two rowers. The main objective was to track the movements of each handle bar and check the time synchronization between rower strokes during the workout. Three telosb equipped with the sensor board presented above were used in the experiment. Two of them were installed in the handlebar of the ergometer. The Z axis was oriented parallel to the pulling direction. The third one was used as a base station, the timestamped data was recollected from the accelerometers at 83 Hz. In Figure 7 the integrated acceleration readings show the velocity measurements of the two sportsman. The local maxima of each timeseries represents a stroke. The observed drift is attributed to the accumulation of errors highlighted by the numerical integration process.

**Figure 8.**
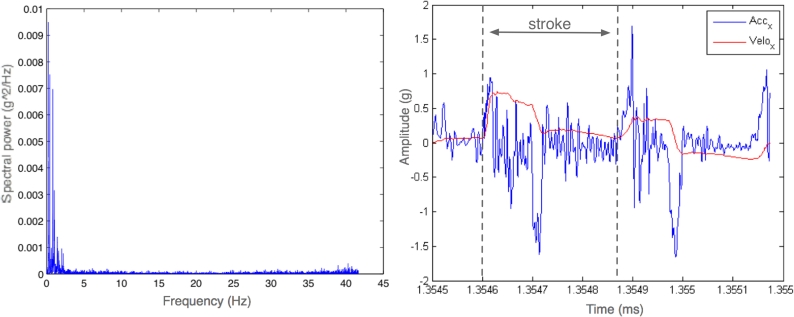
Left: Power spectra of the acceleration readings. Right: Acceleration and velocity time series.

All time series were corrected for instrument response and offset. A spectral analysis of the ergometer data (Figure 8, right) was performed to check the dominant frequencies of the signal. After spectral analysis, the signal were low pass filtered. Specifically, we used a fourth order Butterworth filter with a cutoff frequency of 10 Hz. High frequency background noise were eliminated of the signal. The power spectra of the accelerations registered in Z direction for both ergometers are represented in Figure 8, left.

As observed in Figure 8, the dominant frequencies remain below 5 Hz. The frequency corresponding to the maximum of the spectra is observed at 0.5 Hz. This value can be attributed to the mean cadency of the rower which corresponds, in that case to 30 strokes/minute. These results indicate that time/frequency analysis could be a useful tool rowers cadency evolution monitoring. In Figure 8 (right) the acceleration and the velocity time series of two strokes is presented.

Once the performance of the system in ergometer is proven, the sensors were installed in a boat. Two accelerometers were used in each oar as presented in Figure 4. These sensors were mounted at the end of the oar with X axis pointing towards boat movement direction when the oar was perpendicular to the water and Y axis pointing directly up out of the boat when the oar was perpendicular to the water. The boat sensor was mounted on the hull of the boat to register boat motion.

**Figure 9.**
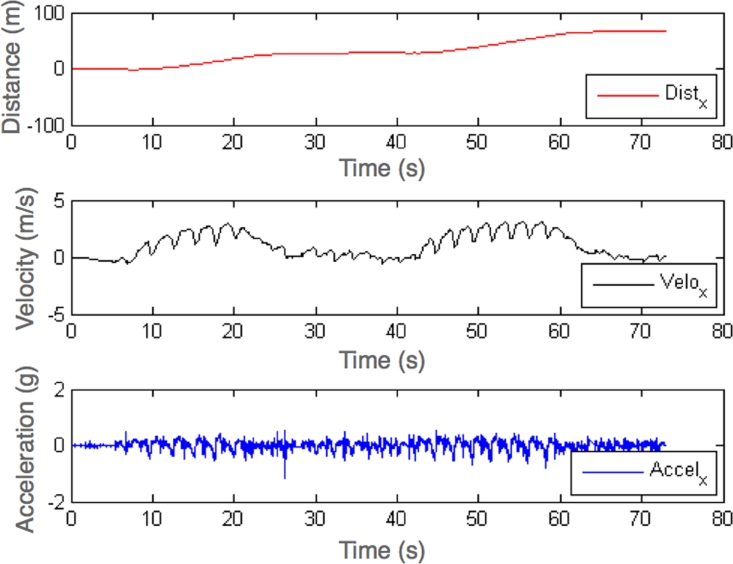
Boat movement.

A series of experiments were performed with this setup. In Figure 9 the motion of the boat during 80 seconds of exercise is presented. The velocity and position were obtained by integration of the acceleration readings. The exercise consisted in going and returning along 40 meters stretch. In Figure 9 middle, the velocity profile evidences the three stages that corresponds to go, turn, and go back. Moreover the ripple observed in the velocity profile shows the effects of the strokes. In Figure 9 top, the boat covered distance is presented.

**Figure 10.**
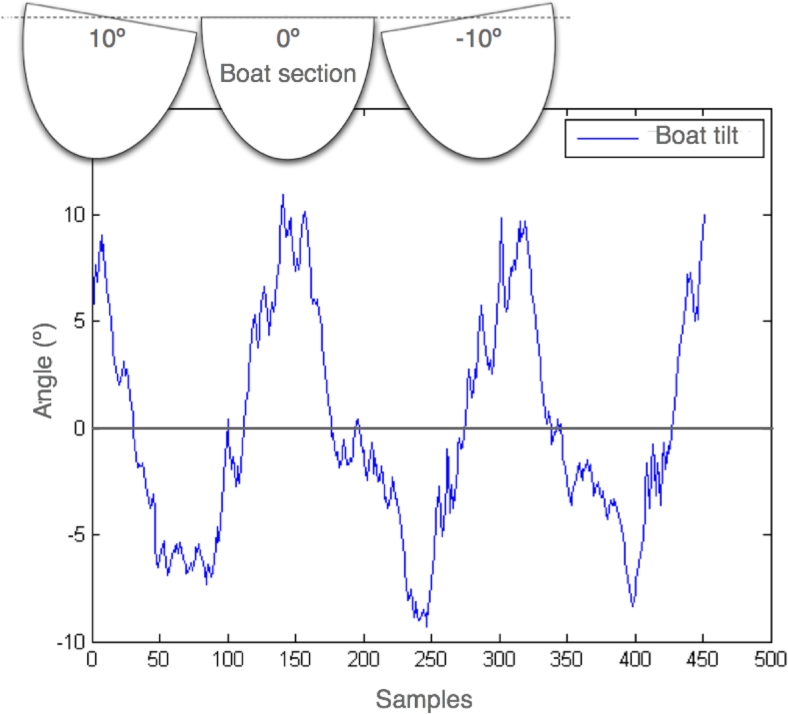
Balancing of the boat.

REMOTE system also permits the study of boat balancing, which is a very important parameter that reflects rower’s technique. In Figure 10 the boat balancing obtained form the boat sensors acceleration readings is presented. On the inset, a scheme of the hypothetical boat tilt is presented showing the associated angles.

**Figure 11.**
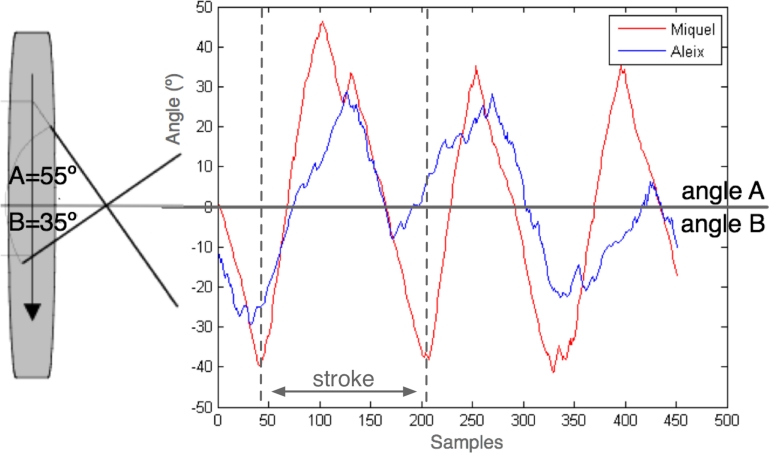
Stroke angles of two sportsman during three strokes.

One of the most important parameters to quantify rowers technique is the stroke angle. Figure 11 (left) show the optimal angles of a stroke (A = 55°, B = 35°). REMOTE system uses the sensors placed at the oars to obtain those angles. In Figure 11 (right) the stroke angles from two rowers during three strokes are presented. For that experiment, two rowers with different experience where selected. Whereas red (Miquel) was a experienced rower and presents a depured technique, beeing his strokes angles close to the optimal angles, blue (Aleix) lacks of depured technique.

**Figure 12.**
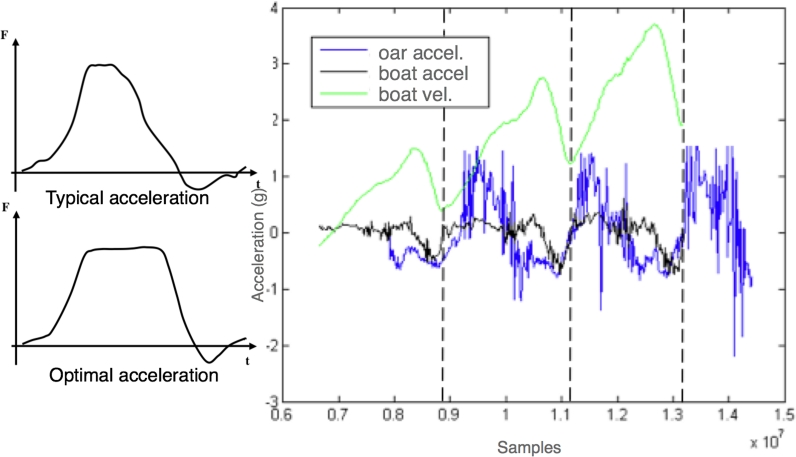
Comparison of boat acceleration, boat velocity, and oar acceleration.

The technique and strength of the rower is compared to the performance of the boat. In Figure 12 (right) rowers oar acceleration, boat acceleration, and boat velocity during three strokes are presented. In Figure 12 (left) the typical and optimum evolution of the oar acceleration are shown. Oar acceleration (Figure 12, right, blue line) resembles the typical profile presented in Figure 12, top, left. Consequently, the trend of the oar acceleration could be used to quantify the technique of the rower.

**Figure 13.**
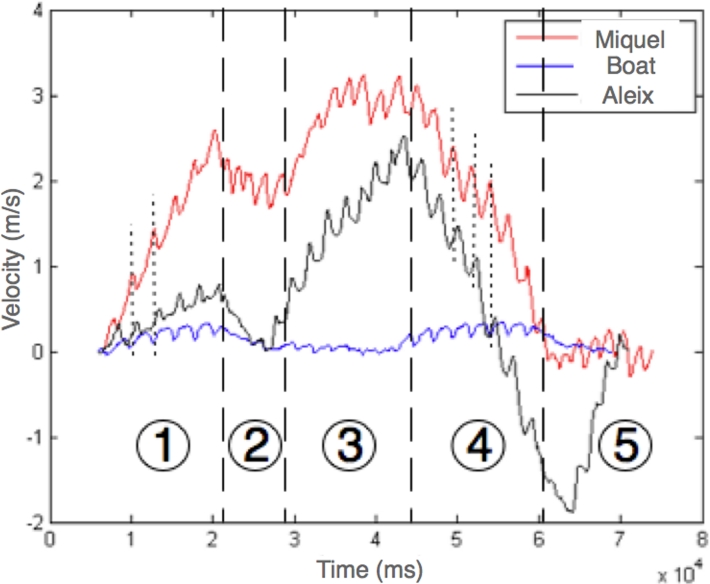
Velocities of the oars and the boat.

In our test we also compared the velocity evolution of two rowers. Figure 13 presents two sportsmen rowing on the same boat reproducing the same go/go back experiment presented above. Note, that the velocity of the boat is also depicted in a blue line. In the Figure 13, 5 stages are clearly evidenced. Where stages 1 and 4 corresponds to covering the stretch, and 2, 3 and 5 corresponds to stopping and turning stages. In stages 1 and 4, the synchronization among the two rowers could be appreciated (note the dotted lines). The higher velocity of Miquel (red line) is attributed to its higher stroke angle as we observed in Figure 11.

**Figure 14.**
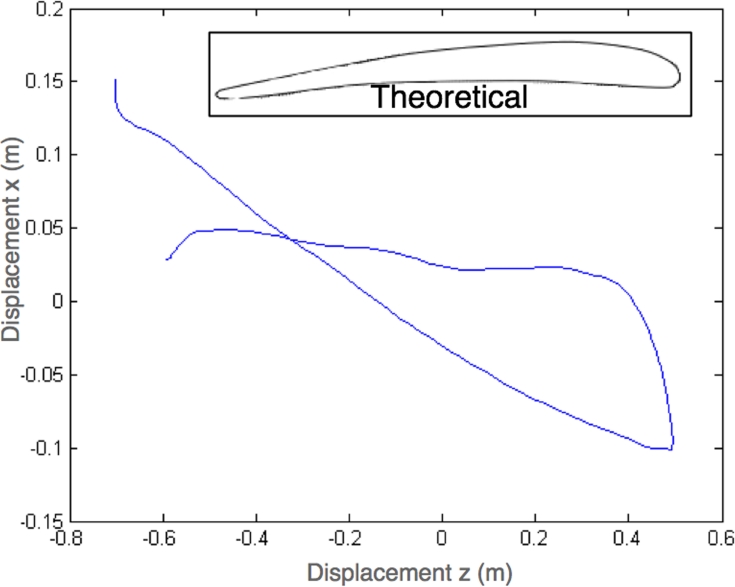
Inset: Theoretical trajectory of a stroke. Trajectory of a stroke obtained with the system.

Finally, the system also permits the study of stroke trajectory. In Figure 14 the trajectory of a single stroke is presented next to the theoretical trajectory. REMOTE provides a good tool to quantify rower techniques. We attribute the observed deviation to mechanical construction of the hand made packaging, unknown original position of the oar, and numerical integration errors.

### 4.2. Remote and RowX Comparison

After comparing the obtained data with the theoretical expected, a feature comparison with RowX is also presented. RowX software provides data form the sensors that are placed around the boat. Thanks to those sensors, RowX is capable of providing the acceleration of the boat in vertical and advancing direction, angles of the oars, speed of the boat, force applied at each oar, and the heart rate. As we have seen REMOTE is capable of obtaining a set of data including most of the RowX data. REMOTE directly provides acceleration of the boat and each oar in the six degrees. This allows us to deduce many types of data, including the data provided by RowX system and other information like the balancing of the boat (Figure 10) and the trajectory of the stroke (Figure 14).

The trajectory of the stroke is the most valuable of the data since it can be used to quantify the rowing technique of the sportsman. Pattern matching algorithms could be applied to evaluate different aspects of the stroke. This could provide information of how the technique changes under different aspects, like stress, tiredness, or even over the time or a certain period of specific training.

The heart rate is not obtained because REMOTE is not equipped with a heart rate sensor. The reason was that we wanted to place all the components inside the oar, but a standard external heart rate sensor could be easily added.

## 5. Conclusions

In this study, a system has been presented to monitor rowing performance based on wireless sensor networks. The system is based on telosb platform and a specifically designed sensor board. The sensor board was equipped with two 3 axis MEMS accelerometers which allows oars and boat motion tracking. We demonstrated that with smart geometrical configuration of the sensors, rotation and translation of the oars and boat can be obtained. Three different tests were performed: laboratory calibration allowed us to become familiar with the accelerometer readings and validate the theory, ergometer tests which helped us to set the acquisition parameters, and on boat tests have shown the potential of this technologies applied to sports.

Wireless sensors and MEMS devices showed to be suitable for sports monitoring due to its reduced size and wireless capabilities. Not limiting athletes’ freedom of motions, and not interfering with their exercise.

Eventually, we can conclude that similar system as RowX can be developed based on REMOTE.
